# Coronary vessel wall MRI at 3.0 T using Time-Resolved Acquisition of Phase-Sensitive DIR (TRAPD): initial results in patients with risk factors for coronary artery disease

**DOI:** 10.1186/1532-429X-15-S1-P26

**Published:** 2013-01-30

**Authors:** Khaled Z Abd-Elmoniem, Ahmed Gharib, Roderic I Pettigrew

**Affiliations:** 1 National Institute of Diabetes and Digestive and Kidney Diseases, National Institutes of Health, Bethesda, MD, USA; 2 National Institute of Biomedical Imaging and Bioengineering, National Institutes of Health, Bethesda, MD, USA

## Background

Technical challenges still hinder coronary wall imaging for routine clinical utilization. These challenges include image degradation due to aperiodic intrinsic cardiac and chest wall respiratory motions and residual motion due to uncompensated heart-rate variability. The purpose of this study was to 1) develop a time-resolved acquisition of phase-sensitive DIR coronary vessel wall MRI technique that overcomes the loss of the orthogonality due to uncompensated residual motions, 2) investigate the associated improvement in coronary wall imaging success rate when compared to that of single image coronary wall imaging, and to 3) assess the ability of the technique to show a difference in vessel wall thickness between healthy subjects and subjects with risk factors for coronary artery disease (CAD).

## Methods

A 3T single-slice time-resolved free-breathing PS-DIR (TRAPD) coronary vessel wall imaging sequence was implemented, validated in a flow phantom. The coronary arterial wall, using 3 to 5 cine-image measurements, were obtained of 26 subjects with at least one Framingham CAD risk factors and 12 healthy subjects without history or risk factors for CAD. Image quality was scored and assessed, wall thickness was automatically measured, and qualitative and quantitative comparisons were made between TRAPD and conventional single-image wall measurements.

## Results

**Figure 1:**
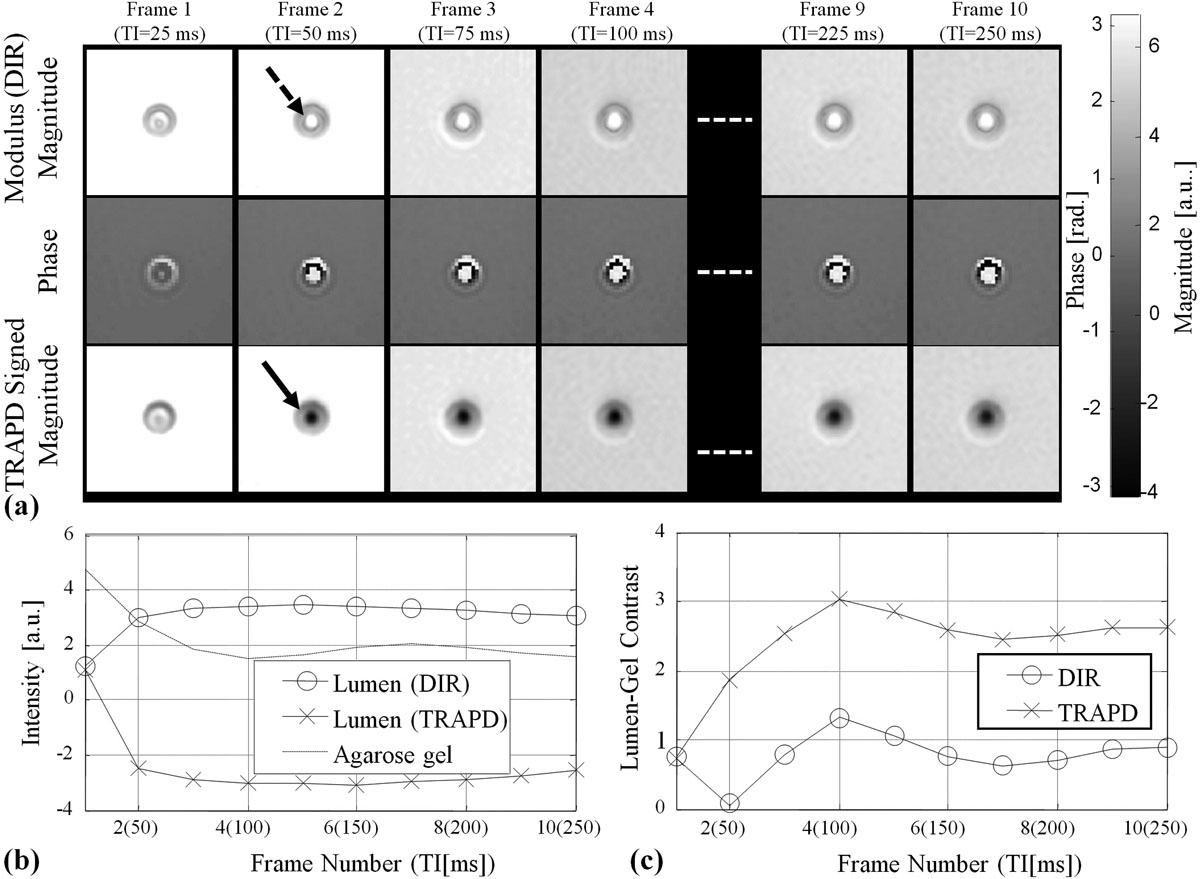
TRAPD Phantom signal intensity and contrast using incremental TI ranging from 25 ms to 250 ms, a) modulus (DIR), phase, and TRAPD signed-magnitude images, b) signal intensity inside and outside the lumen, c) DIR and TRAPD tissue-lumen contrasts.

**Figure 2:**
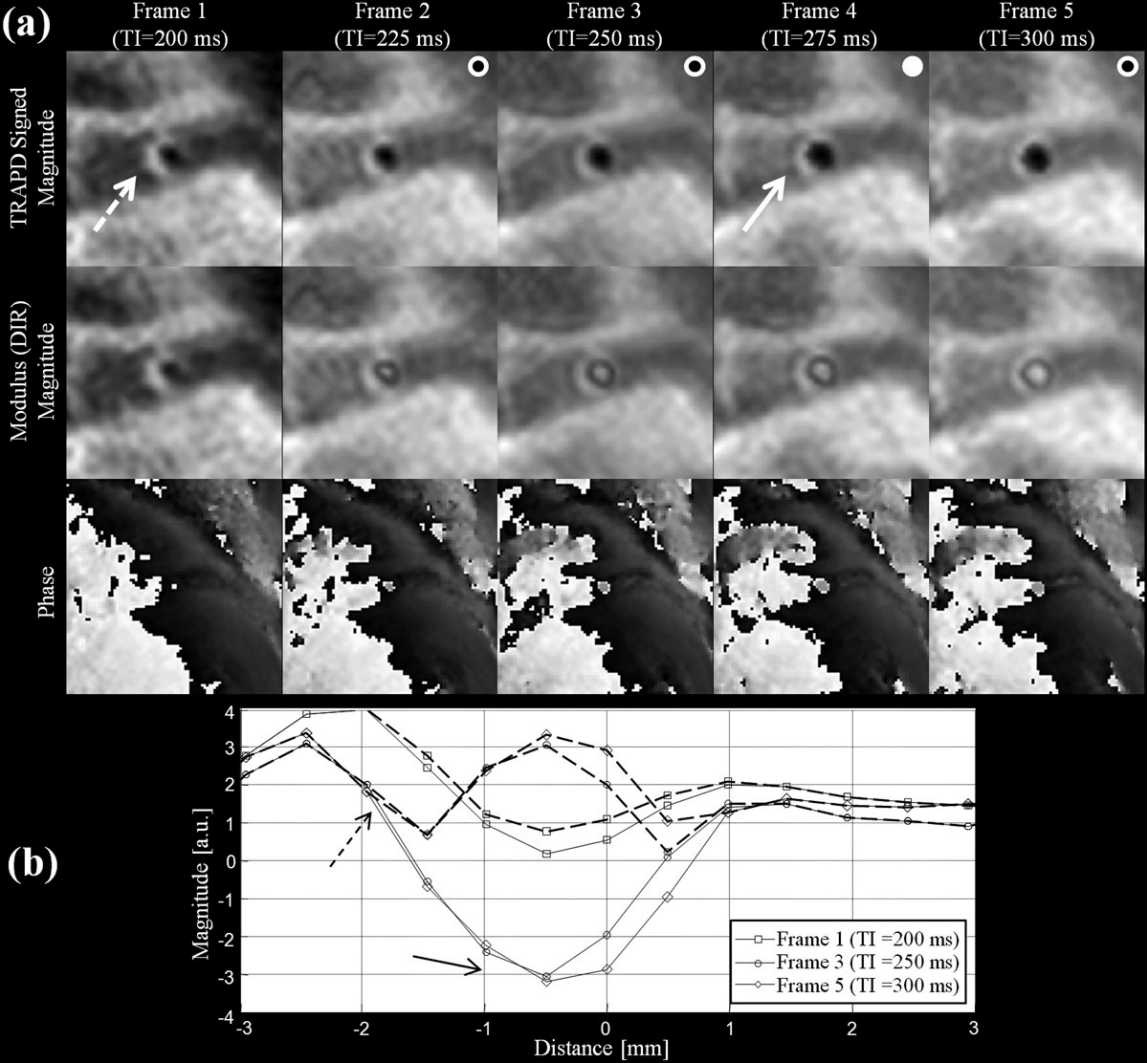
a) TRAPD (top row) and conventional DIR modulus images (second row) of a healthy subject. White circles denote the frames with good or excellent quality. Filled circle denotes the frame with the thinnest wall thickness measurement. b) Line intensity profiles across the coronary vessel wall and lumen at different time frames using TRAPD (solid lines) and DIR (broken lines).

Time resolved coronary vessel wall imaging using TRAPD successfully restored the negative polarity of lumen signal and enhanced lumen-wall contrast in the cine images in flow-phantom (Figure 1), and in both normal and subjects with coronary risk factors (Figure 2). The acquisition and utilization of the additional frames increased the cumulative success rate of acquiring at least one adequate-quality image from 76% in single-image acquisitions to over 90% when five frames were acquired. Utilizing multiple consecutive frames in calculations achieved more separation between the normals' and patients' mean wall thickness values, and with more precision demonstrated by a narrower standard deviation. The difference in vessel wall thickness between the two subject groups was statistically significant (p<0.05) when using TRAPD (1.07 mm vs. 1.46 mm; 36% increase) compared to single-frame DIR imaging (1.24 mm vs. 1.55 mm; 25% increase).

## Conclusions

Fast time-resolved phase-sensitive DIR imaging was implemented to improve coronary arterial wall imaging by increasing the success rate of obtaining good to excellent quality images inclusive of slice vessel orthogonality (i.e., slices orthogonal to longitudinal axis of the vessel). This has resulted in vessel wall thickness measurements that show a more distinct difference between normal and patient populations.

## Funding

National Institutes of Health

